# Ultraviolet A-Induced Cathepsin K Expression Is Mediated via MAPK/AP-1 Pathway in Human Dermal Fibroblasts

**DOI:** 10.1371/journal.pone.0102732

**Published:** 2014-07-21

**Authors:** Qingfang Xu, Wei Hou, Yue Zheng, Chen Liu, Zijian Gong, Chun Lu, Wei Lai, Howard I. Maibach

**Affiliations:** 1 Department of Dermato-Venereology, the Third Affiliated Hospital of Sun Yat-sen University, Guangzhou, PR China; 2 Department of Dermato-Venereology, the First Teaching hospital of Xinjiang Medical University, Urumqi, PR China; 3 Department of Dermatology, School of Medicine, University of California San Francisco, San Francisco, California, United States of America; National Cancer Centre, Singapore

## Abstract

**Background:**

Cathepsin K (CatK), a cysteine protease with the potent elastolytic activity, plays a predominant role in intracellular elastin degradation in human dermal fibroblasts (HDFs), and contributes to solar elastosis. In previous studies, CatK expression was downregulated in photoaged skin and fibroblasts, but upregulated in acute UVA-irradiated skin and fibroblasts. The underlying mechanisms regulating UVA-induced CatK expression remain elusive.

**Objective:**

This study investigates mechanisms involved in the regulation of UVA-induced CatK expression in HDFs.

**Methods:**

Primary HDFs were exposed to UVA. Cell proliferation was analyzed using a colorimetric assay of relative cell number. Quantitative real-time RT-PCR and Western blot were performed to detect CatK expression in HDFs on three consecutive days after 10 J/cm^2^ UVA irradiation, or cells treated with increasing UVA doses. UVA-activated MAPK/AP-1 pathway was examined by Western blot. Effects of inhibition of MAPK pathway and knockdown of Jun and Fos on UVA-induced CatK expression were also measured by real-time RT-PCR and Western blot.

**Results:**

UVA significantly increased CatK mRNA and protein expression in a dose-dependent manner. UVA-induced CatK expression occurred along with UVA-activated phosphorylation of JNK, p38 and Jun, UVA-increased expression of Fos. Inactivation of JNK and p38MAPK pathways both remarkably decreased UVA-induced CatK expression, which was suppressed more by inhibition of JNK pathway. Furthermore, knockdown of Jun and Fos significantly attenuated basal and UVA-induced CatK expression.

**Conclusion:**

UVA is capable of increasing CatK expression in HDFs, most likely by activation of MAPK pathway and of AP-1, which has been shown to be the case for matrix metalloproteinases. As current strategies for selecting anti-photoaging agents focus on their ability to decrease MMPs' expression through inhibiting UV- activated MAPK pathway, future strategies should also consider their effect on CatK expression.

## Introduction

Photoaging is histologically characterized by reduced content of dermal collagen fibers and accumulation of dystrophic elastotic material, the latter commonly termed solar elastosis [1]. Studies on photoaging mainly focus on decreased collagen caused by increased matrix metalloproteinases (MMPs)' degradation, but fewer consider solar elastosis. Although the pathogenesis of solar elastosis has been considered mainly because of overproduction and/or decreased degradation of elastic fibers [2], the exact mechanism remains elusive. Elastase secreted by infiltrating neutrophils is often reported to be a major player in elastin degradation [3]. MMP-2, MMP-7, MMP-9 and MMP-12 also have elastolytic activity [4], [5]. All these proteases are secreted to mediate elastin degradation in the extracellular space. Sustained increase of their expression has been shown in the UV-exposed skin [4], [6], yet they can't counteract the increased synthesis of elastin in solar elastosis.

Cathepsin K (CatK) is a member of the cystein protease family with potent elastolytic and collagenolytic activity, which contributes to maintenance of the extracellular matrix homeostasis in tissues such as the bone, lung, synovia and skin [7]–[10]. In contrast to the extracellular elastolytic proteases, CatK degrades internalized elastin in the lysosomes of dermal fibroblasts, and plays a predominant role in the intracellular elastin degradation [11]. Intracellular and extracellular elastin degradations are likely inter-dependent and may act in concert. We recently reported that CatK expression of mRNA and protein is decreased in photoaged skin in vivo and fibroblasts in vitro [12]. Decreased CatK expression probably leads to diminished elastin degradation and contributes to solar elastosis. However, UVA upregulates CatK expression in acute UVA-irradiated dermal fibroblasts and explant skin [11]. Little is known about the mechanisms whereby UVA induces expression of CatK in human dermal fibroblasts.

UVA can activate mitogen-activated protein kinase (MAPK) pathway. Its activation leads to activator protein-1(AP-1) induction, which regulates the transcription of MMP genes [13]. Moreover, MAPK/AP-1 pathway mediates CatK expression induced by different stimuli in various cells [14]–[16]. In articular chondrocytes, CatK is enhanced by the N-terminal telopeptide of collagen type II via the activation of p38MAP kinase [14]. p38MAP kinase is also essential for the induction of CatK gene expression by RANKL in osteoclasts [17]. Furthermore, AP-1 stimulates the CatK promoter in RAW 264.7cells [18]. We hypothesize that UVA-induced CatK expression in human dermal fibroblasts, similar to MMPs, may also be mediated by MAPK/AP-1 pathway.

This study investigates whether MAPK/AP-1 pathway is involved in the regulation of UVA-induced CatK expression in human dermal fibroblasts, by inhibition of JNK and p38MAPK pathways and knockdown of Jun and Fos.

## Materials and Methods

### Ethics Statement

Parents on behalf of their children enrolled signed an informed consent form. They were told about our research objectives and their privacy and anonymity were protected. The consent procedure was conducted according to the principles expressed in the Declaration of Helsinki. Both the consent procedure and our study were approved by the Clinical Research Ethics Committee at the Third Affiliated Hospital of Sun Yat-sen University, Guangzhou, China (No: [2010]2-22).

### Reagents

All inhibitors (SP600125, a JNK-specific inhibitor; SB203580, a p38-specific inhibitor) were purchased from Calbiochem (San Diego, CA). Anti-human CatK antibody (#19027) was obtained from Abcam(Cambridge, MA, USA). Primary antibodies against phospho-JNK (P-JNK, #9251), JNK(#9258), phospho-p38 (P-p38, #4511), p38(#9212), phospho-Jun (P-Jun, #3270), Jun(#9165), phospho-MAPKAPK-2(P-MAPKAPK-2, #3316), Fos(#2250), GAPDH(#2118) and anti-rabbit HRP-conjugated secondary antibody(#7074) were purchased from Cell Signaling Technology (Boston, MA, USA). siRNA targeting Jun (SASI_HsO2_00333461, sense strand: 5′- GAUGGAAACGAC CUU CUA UdTdT -3′, anti-sense strand: 5′-AUAGAAGGUCGUUUCCAUCdTdT-3′), Fos (SASI_HsO1_00115496, sense strand:5′-CACACAUGAUGUUUGACGAdTdT-3′, anti-sense strand:5 ′-UCGUCAAACAUCAUGUGUGdTdT-3′), and non-targeting control siRNAs were obtained from Sigma-Aldrich (Shanghai, China). Lipofectamine RNAiMAX transfection reagent and Opti-MEM were bought from Invitrogen and Gibco (Grand Island, NY, USA) respectively.

### Cell culture

Human dermal fibroblasts isolated from circumcised foreskins aged from 6 to 9 years were cultured in Dulbecco's modified Eagle's media (DMEM; Gibco, Grand Island,NY, USA) supplemented with 100 U/mL penicillin/streptomycin and 10% fetal bovine serum(FBS; Gibco). They were grown at 37°C in a humidified 5% CO2 atmosphere and used for experiments up to passage number ten. Repeat experiments were performed using cells from different donors.

### UVA irradiation and CCK-8 assay

Philips UVA TL10RS lamps with an emission spectrum between 320 and 400 nm were the UVA source. UVA dose was measured using a UVA radiometer (Sigma, Shanghai, China). Briefly, cells in culture plates were rinsed twice with phosphate-buffered saline (PBS).Then irradiations were performed with cells under a thin layer of PBS. Mock-irradiated control cells under PBS were put in clean bench, and covered with foil. Immediately after irradiation, PBS was replaced with fresh culture medium, and cells were incubated for the indicated time.

A cell counting kit-8(CCK-8, Dojindo, Japan) assay was used to determine cell viability. It is based on the dehydrogenase activity detection in viable cells. The formazan dye generated by dehydrogenases absorbs light of a wavelength of 450 nm. The amount of the formazan dye in cells is directly proportional to the number of living cells. In brief, cells were incubated in 96-well culture plates overnight, and then treated with UVA irradiation or inhibitors. After incubation in fresh culture medium for 24 h, 10 µL CCK-8 solution was added. Cells were incubated at 37°C for 4 h. Absorbance was analyzed at 450 nm using an ELISA reader.

### Treatment of MAPK inhibitors

SP600125 and SB203580, which inhibit phosphorylation of Jun and MAPKAPK-2 respectively [19], [20], were chosen to block JNK and p38 activation accordingly. They were dissolved in dimethyl sulfoxide (DMSO) (10 mM stock solution), and diluted with culture media at the indicated concentrations. Silvers *et al* [21] noted that the combination of 250 kJ/m^2^ UVA with low doses of SP600125 resulted in dramatic morphologic changes and increased cell death. Therefore, we used CCK-8 assay to determine the final SP600125 concentration. 10 µM SB203580 concentration was used according to manufacture's instruction. Cells were pretreated with inhibitors for 1 h before UVA irradiation, and incubated with inhibitors till harvesting.

### Transient silencing of Jun and Fos

5×10^4^ primary fibroblasts per well were seeded in 6-well plates overnight, then were transfected at 50–70% confluence with 50 nM Jun siRNA and 50 nM Fos siRNA using Lipofectamine RNAiMAX transfection reagent according to the manufacturer's protocol. The silencing efficiency of siRNA was determined by detecting the expression of Jun and Fos at the mRNA and protein levels in transfected cells. At 24 h after transfection, medium was replaced with PBS, cells were irradiated by 10 J/cm^2^ UVA or mock treated before transferred into fresh culture medium for an additional 48 h. RNA and protein were then harvested for quantitative real-time RT-PCR and Western-blot analysis for CatK.

### RNA extraction and quantitative real-time RT-PCR

Total RNA was isolated using Trizol (Invitrogen, Germany) according to the manufacturer's instruction and quantified spectrophotometrically. One microgram of total RNA from each sample was subjected to first-strand cDNA synthesis using a PrimeScript RT reagent kit (TaKaRa Bio Inc.) at 37°C for 15 min and 85°C for 5 s. Real-time PCR amplifications were performed on ABI PRISM 7500 Sequence Detection System with SYBR Premix Ex Taq TM (TaKaRa Bio Inc.). Primers for PCR reaction were: CatK, 5′-GTCTGAGAATGATGGCTGTGGA-3′ (forward) and 5′- CATTTAGCTGCCTTGCCTGTTG-3′(reverse) with a product size of 150 bp; Jun, 5′-GAAGTGTCCGAGAACTAAAG-3′(forward), 5′-AAAAGTCCAACGT-TCCGTTC-3′ (reverse) with a product size of 189 bp; Fos, 5′- TCTCCAGTGCCAACTTCATT-3′(forward), 5′-GTGTATCAGTCAGCTCCCTC- 3′(reverse) with a product size of 330 bp; GAPDH, 5′-GCACCGTCAAGGCTGAGA-AC-3′ (forward) and 5′-TGGTGAAGACGCCAGTGGA- 3′ (reverse) with a product size of138 bp. The PCR cycles were 95°C for 30 s, followed by 40 cycles of 95°C for 5 s and 60°C for 20 s. Gene expression was normalized to the level of GAPDH in a given sample, and was quantified using the comparative Ct method. ΔCt (Ct target- Ct endogenous control) and ΔΔCt(ΔCt target-ΔCt calibrator) were determined according to the ABI Prism 7500 sequence detection system user guide. Relative expression levels of target genes were calculated using 2^−ΔΔCt^ as the fold-increase over controls [18].

### Western-blot analysis

Total cellular protein was extracted with the total protein extraction kit (KeyGen Biotech, Nanjing, China). Protein concentration was determined using a BCA protein assay kit (Key-Gen Biotech). Equal amounts of protein were analyzed using 10%SDS–PAGE, and transferred to a PVDF membrane. Anti- CatK and anti-GAPDH antibodies were diluted at 1∶500 and 1∶4000 respectively. Primary antibodies against P- JNK, JNK, P-p38, p38, P-Jun, Jun, P-MAPKAPK-2 and Fos were diluted at 1∶1000, and incubated with membranes overnight at 4°C. Following subsequent incubation with a secondary antibody, blots were visualized with enhance chemiluminescence (Millipore, Billerica, MA, USA). Bands on X-ray film were scanned with GS-800 Calibrated Densitometer (Bio-rad, USA). The intensity of bands was quantified with Quantity one software. All values were normalized to the corresponding GAPDH.

### Statistical analysis

Results were representative of at least three independent experiments. All values were expressed as means ± SD. Statistical significance was determined by a one-way analysis of variance (ANOVA), followed by the LSD test. P values of <0.05 were considered statistically significant.

## Results

### UVA induces CatK mRNA and protein expression in a dose-dependent manner

**Figure 1:**
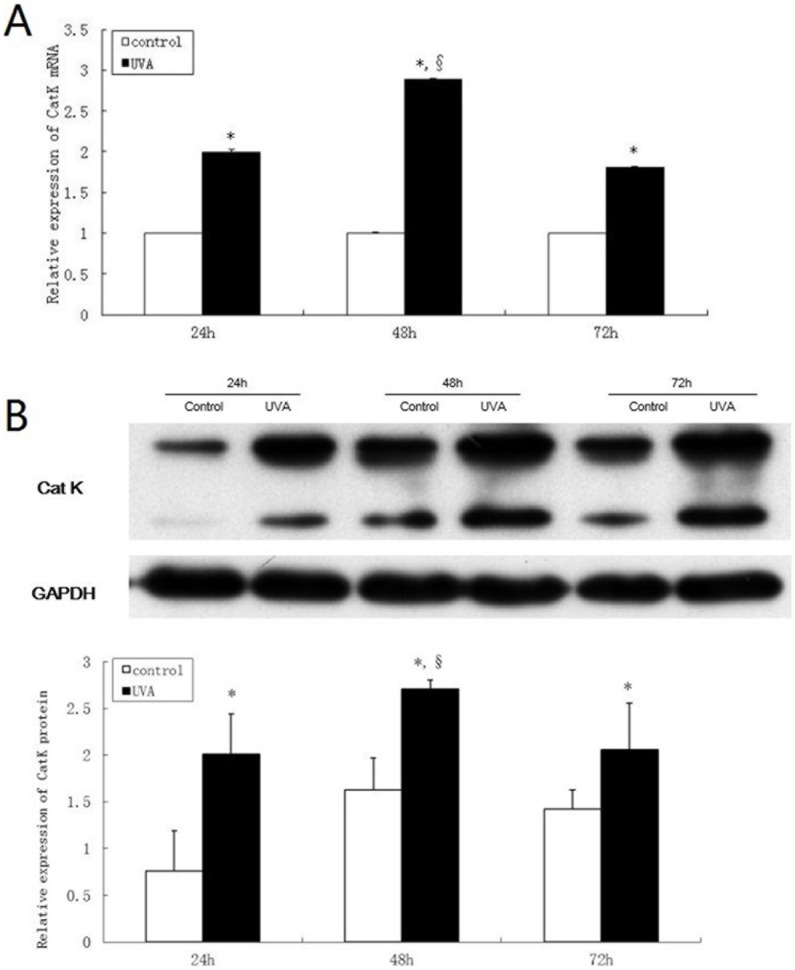
UVA increases CatK expression at both mRNA and protein levels for three consecutive days in human dermal fibroblasts. Cultured fibroblasts were irradiated with sham and 10/cm^2^ UVA. Cells were harvested on three consecutive days after irradiation for RNA and protein extraction. (A) Total cellular RNA was analyzed by real-time RT-PCR for CatK. (B) Cell lysates were analyzed by Western blot with anti-CatK antibody. The blot was reprobed with anti-GAPDH to confirm equal loading. Bar graphs represent the relative band intensities (mean±SD from three independent experiments). *P<0.05 vs. untreated control, §P<0.05 vs. UVA-treated groups 24 h and 72 h after irradiation.

**Figure 2:**
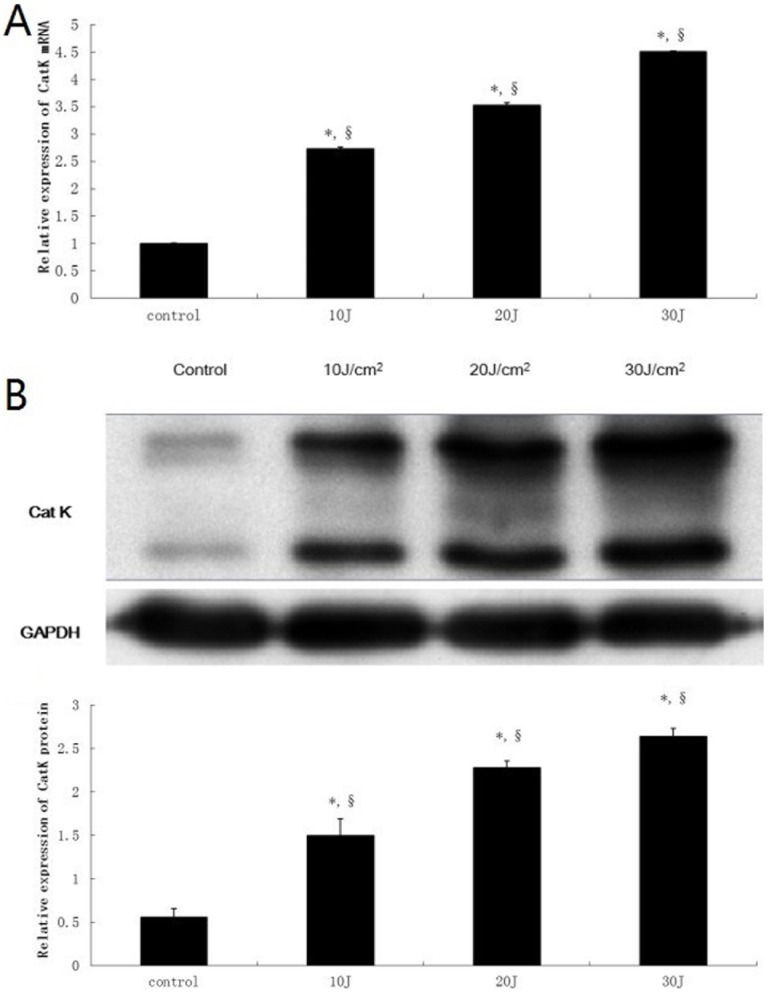
UVA induces CatK expression in a dose-dependent manner. Cultured fibroblasts were mock irradiated or irradiated with 10/cm^2^ UVA, 20 J/cm^2^ UVA and 30 J/cm^2^ UVA. Cells were harvested 48 h after UVA irradiation for total RNA and protein extraction. (A) Quantitative analysis of CatK mRNA level with real-time RT-PCR. (B) Analysis of CatK protein level by western blot using anti-CatK antibody. Blots were reprobed with anti-GAPDH to confirm equal loading. Bar graphs represent the relative band intensities (mean±SD from three independent experiments). *P<0.05 vs. untreated control, §P<0.05 vs. both other dose-treated groups.

To determine whether UVA stimulates CatK expression in human dermal fibroblasts, we studied mRNA and protein expression of CatK. Twenty-four hours after irradiation, doses of up to 10 J/cm^2^ UVA did not change cell morphology, and resulted in more than 90% viable cells. Therefore, 10 J/cm^2^ UVA exposure was used in most of the following experiments. Irradiation of cultured dermal fibroblasts with 10 J/cm^2^ UVA increased both mRNA and protein expression of CatK for three consecutive days with a maximum 48 h after irradiation (Fig.1). Furthermore, CatK mRNA and protein expression were induced by UVA treatment in a dose-dependent manner (Fig.2). Pro- (upper band, 37 kd) and active (lower band, 25 kd) forms of CatK were detected with Western-blot analysis, and UVA increased the expression of both.

### UVA activates MAPK pathway and increases the expression of P-Jun and Fos

**Figure 3:**
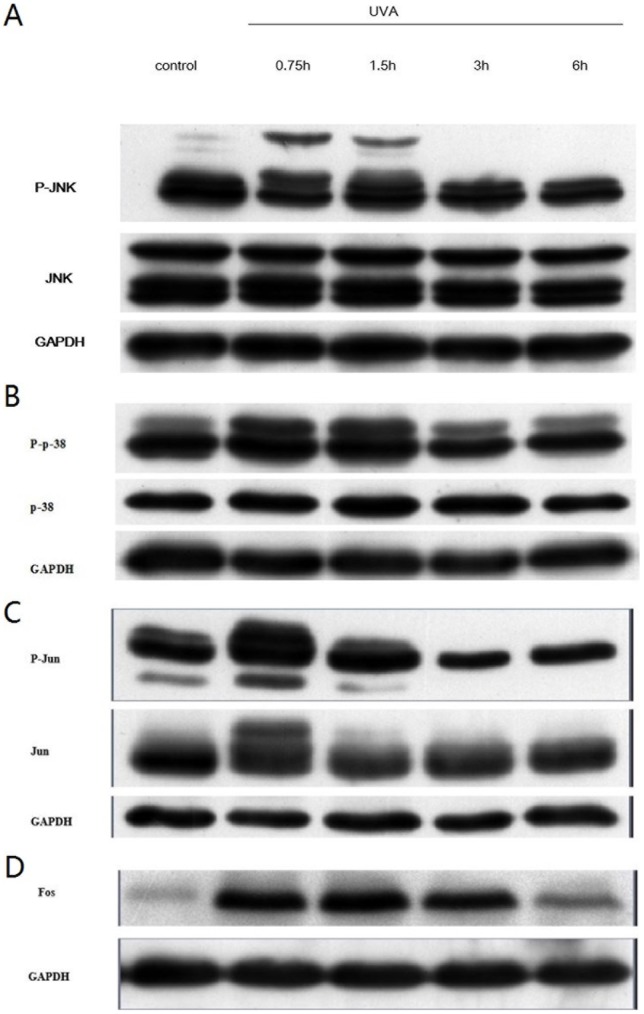
Time courses for UVA-induced MAPK activation and AP-1 activity. Cells were mock-irradiated or irradiated with 10 J/cm^2^ UVA and harvested at the indicated time points. Western blot analysis was performed and the representative data of three independent experiments were shown. Figure showed the expression of (A) JNK; (B) p38; (C) Jun; (D) Fos.

Since UVA doesn't activate ERK in human skin fibroblasts [22], we didn't study the activation of this pathway. Analysis of Western-blot was performed to detect the time courses of P-JNK, P-p38, P-Jun and Fos, beginning 0.75 h and spanning 6 h after UVA irradiation. As shown in Fig.3A, B and D, P-JNK, P-p38 and P-Jun were significantly induced between 0.75 h∼1.5 h, and subsequently declined, whereas the level of total JNK and p38 MAPK was unaffected. Increased expression of Fos was detected at 0.75 h and persisted till 6 h after treatment, with the maximal expression between 0.75 h and 1.5 h (Fig.3D). In addition, expression of Jun was also increased at 0.75 h after irradiation (Fig.3C).

### Inhibition of MAPK pathway decreases UVA-induced CatK expression

UVA activates MAPK pathway and increases CatK expression. Therefore, we examined the relationship between MAPK activation and UVA-induced CatK expression in human dermal fibroblasts. First, we evaluated effects of pharmacologic MAPK cascade inhibitors on UVA-induced MAPK activation. Twenty-four hours after the combined treatment of 10 J/cm^2^ UVA irradiation and SP600125 at concentration of 1 µM or higher, a significant decrease in cell viability was observed. Therefore, we used 800 nM SP600125 in the following experiments.

**Figure 4:**
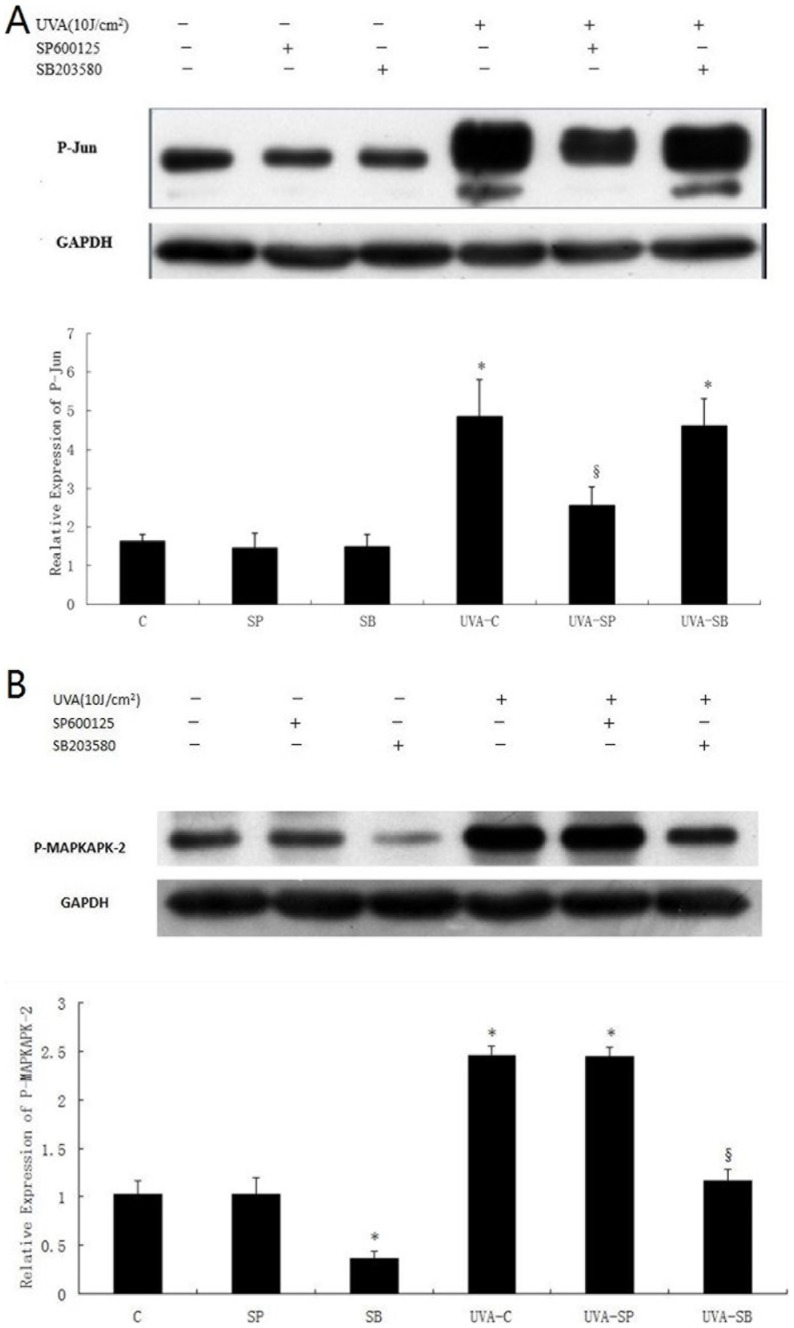
Inhibitory effects of SP600125 and SB203580 on UVA-activated MAPK pathway. Cells were pretreated with 800 µM SB203580 for 1 h, mock-irradiated or irradiated with 10 J/cm^2^ UVA, and then incubated in culture medium supplemented with MAPK inhibitors for 1.5 h. Fifteen micrograms of total cellular lysate was electrophoresed on 10% SDS polyacrylamide gels, transferred to a PVDF membrane, and immunodetected using optimal primary and secondary antibody concentrations. Western blot data are representative of three independent experiments. (A) Effects of SP600125 and SB203580 on JNK activity through detection of changes in Jun phosphorylation; (B) Effects of SP600125 and SB203580 on p38 activity through detection of changes in MAPKAPK-2 phosphorylation. *P<0.05 vs. untreated control, §P<0.05 vs.UVA-treated control.

As shown in (Fig.4A and 4B), treatment of fibroblasts with 800 nM SP600125 resulted in a significant decrease of UVA-induced P-Jun without affecting p38 activation, implying that JNK activation could be effectively and specifically inhibited by 800 nM SP600125. Also, 10 µM SB203580 was found to significantly suppress expression of UVA-stimulated phospho-MAPKAPK-2, whereas activation of JNK was not altered, suggesting that p38 activation could be selectively inhibited.

**Figure 5:**
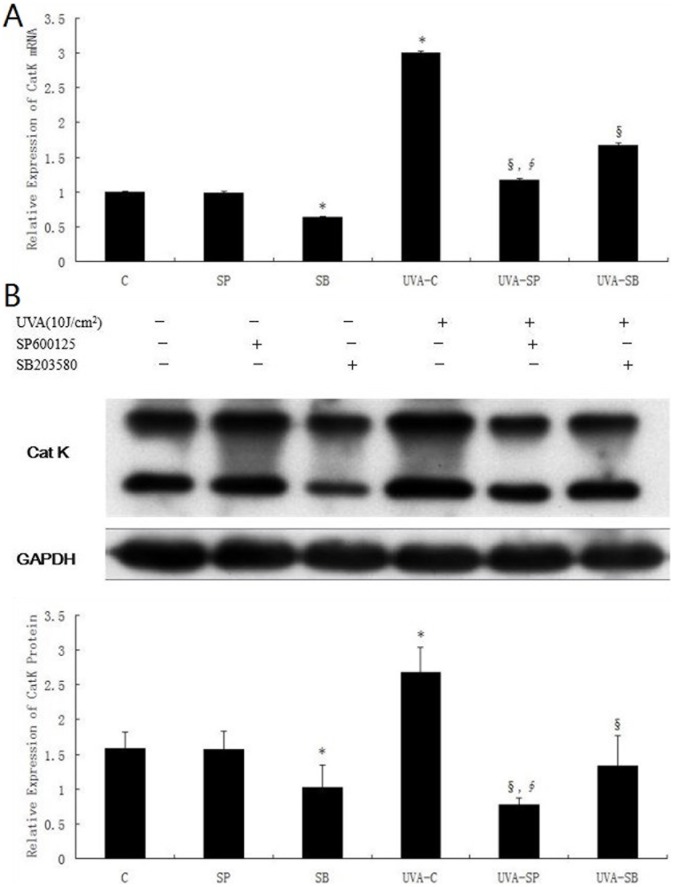
Inhibitory effects of SP600125 and SB203580 on UVA-induced CatK expression. Cells were pretreated with 800 µM SB203580 for 1 h, mock-irradiated or irradiated with 10 J/cm^2^ UVA, and then incubated in culture medium supplemented with MAPK inhibitors for 48 h. (A) Effects of SP600125 and SB203580 on UVA-induced CatK mRNA expression. Total cellular RNA was isolated at 48 h after irradiation, and was analyzed by real-time RT- PCR for CatK. (B) Effects of SP600125 and SB203580 on UVA-induced CatK protein expression. Total cellular protein was harvested at 48 h after irradiation. Cell lysates were analyzed by Western blot with anti-CatK antibody. Blots were reprobed with anti-GAPDH to confirm equal loading. Bar graphs represent the relative band intensities (mean±SD from three independent experiments). *P<0.05 vs. untreated control, §P<0.05 vs. UVA-treated control, ∮P<0.05 vs. UVA and SB203580-treated control.

Next, we investigated effects of MAPK inhibitors on UVA-induced CatK expression. Analysis by real-time RT-PCR revealed that treatments with 800 nM SP600125 and 10 µM SB203580 significantly attenuated UVA-stimulated CatK mRNA expression by approximately 61% and 44.3% respectively, compared with UVA-treated control (P<0.05)(Fig.5A). Furthermore, western blot showed that SP600125 and SB203580 also obviously decreased UVA-induced upregulation of CatK protein by approximately 71.3% and 50.9% respectively, compared with UVA-exposed control (P<0.05) (Fig.5B). Unlike treatment of SP600125, that of SB203580 was found to inhibit mRNA and protein expression of CatK in non-UVA-treated cells. In addition, SP600125 was more effective in suppressing both mRNA and protein levels of UVA-induced CatK than SB203580.

### Knockdown of Jun and Fos by siRNA reduces UVA-induced CatK expression

**Figure 6:**
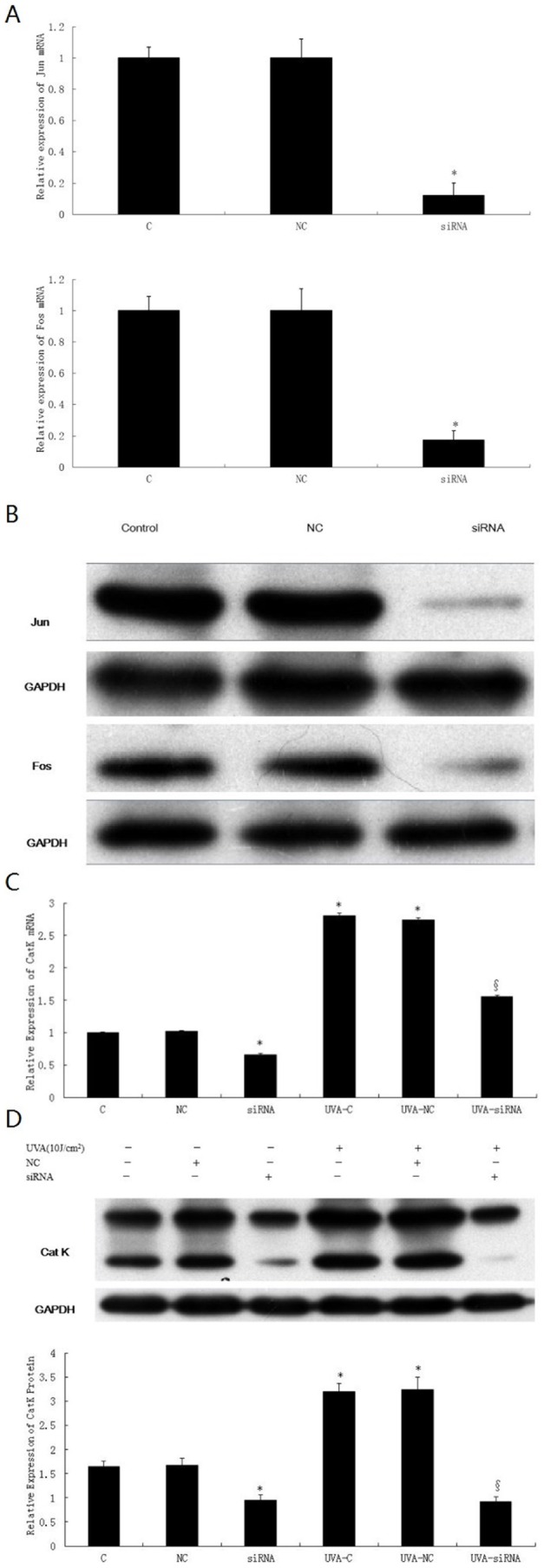
effect of Jun and Fos siRNA on UVA-induced CatK expression. Cells were cotransfected at 50–70% confluence with 50 nM Jun siRNA and 50 nM Fos siRNA, or 50 nM non-targeting control siRNA (NC) according to the manufacturer's protocol. At 24 h following transfection, the medium was replaced with PBS, cells in control (C), non-targeting-siRNA transfected control (NC) and Jun and Fos siRNA transfected group (siRNA) were not irradiated, whereas cells in UVA-C, UVA-NC and UVA-siRNA were irradiated by 10 J/cm^2^ UVA, and then incubated with fresh culture medium for an additional 48 h. (A) mRNA level of Jun and Fos in their siRNA-transfected fibroblasts. (B) protein level of Jun and Fos in their siRNA-transfected fibroblasts.(C) Effect of Jun and Fos siRNA on UVA-induced CatK mRNA expression. Total cellular RNA was isolated at 48 h after irradiation, and was analyzed by real-time RT-PCR for CatK. (D) Effect of Jun and Fos siRNA on UVA-induced CatK protein expression. Total cellular protein was harvested at 48 h after irradiation,then was analyzed by Western blot with anti-CatK antibody. *P<0.05 vs. untreated control, §P<0.05 vs. UVA-treated control.

Activation of MAPK pathway increases AP-1 activity. Therefore, we are interested in determining whether UVA-induced CatK expression is also mediated by AP-1.We transiently cotransfected fibroblasts with Jun siRNA and Fos siRNA to decrease AP-1 expression. Transfection of siRNA was confirmed to markedly reduce the expression of Jun and Fosat the mRNA and protein levels in transfected cells, compared with untreated or non-targeting siRNA-treated controls (P<0.05). Finally, we studied the effect of knockdown of Jun and Fos on UVA-induced CatK expression. By analysis of real-time RT-PCR and Western-blot, we noted that cotreatment of Jun siRNA and Fos siRNA significantly attenuated UVA-stimulated expression of CatK mRNA and protein by approximately 44.6% and 71.2% respectively, compared with UVA-treated control (P<0.05) (Fig 6C, 6D). Moreover, siRNA transfection remarkably reduced basal CatK expression. In contrast, CatK expression in UVA-treated or non-UVA-treated fibroblasts was not changed by non-targeting siRNA.

## Discussion

Solar elastosis is a hallmark of photoaged skin. It has been found to be closely associated with proteases, such as elastases, MMPS and CatK [11], [23], [24], [25]. CatK has recently been reported to play a prominent role in intracellular elastin degradation in comparison with extracellular elastolytic activity of elastases and MMPs [11]. MAPK/AP-1 pathway can be activated by UVA and regulates MMPs expression in human dermal fibroblasts [13]. Thus, we are interested in whether UVA-induced CatK expression in human dermal fibroblasts is also mediated via this pathway.

CatK is strongly expressed in lysosomes of dermal fibroblasts [7]. We studied effect of UVA irradiation on CatK expression in dermal fibroblasts. Our data demonstrated that UVA increased CatK expression for three consecutive days (Fig.1) with maximal induction on the second day, suggesting that single UVA-irradiation may induce CatK expression transiently, two days later CatK expression begins to restore. Moreover, UVA dose-dependently enhanced CatK expression (Fig.2). Our finding confirms the previous study that UVA up-regulates CatK expression both in fibroblasts in vitro and in skin in vivo [11]. However, we recently reported that CatK expression was decreased in photoaged human skin and fibroblasts [12]. Since elastin and fibrillin expression in sun-damaged skin is increased several-fold [26], we hypothesize that enhanced CatK expression early after UVA irradiation may be a protective mechanism, which helps degrade increased elastin synthesis. Along with photoaging, UV-induced mitochondrial DNA deletion [27], acceleration of decorative telomeres and DNA damage may result in the decreased expression of CatK [28]–[30], which further causes solar elastosis. Regardless, the exact mechanisms about the regulation of CatK expression in UVA – irradiated fibroblasts remain elusive and need to be further studied.

UVA activates JNK and p38MAPK pathways [13], which are specifically inhibited by SP600125 and SB203580 respectively [20], [31]. Our study confirmed that 800 nM SP600125 and 10 µM SB203580 effectively and selectively suppressed UVA-induced activation of MAPK pathway (Fig.4A and 4B). Inactivation of JNK pathway was shown to attenuated UVA-stimulated CatK expression from transcription to protein levels more significantly than that of p38MAPK pathway (Fig.5A and 5B), indicating that UVA-induced CatK expression is regulated by both JNK and p38MAPK pathways, probably more by JNK pathway. Our finding is consistent with other studies. p38MAPK pathway was previously reported to be required for induction of CatK mRNA by free cholesterol accumulation in mouse peritoneal macrophages[15]. Furthermore, both CatK gene expression and promoter activity in osteoclasts were stimulated by p38MAPK activation [17], [32]. In addition, tumor necrosis factor alpha stimulated CatK activity via JNK activation in endothelial cells [16]. However, Anke Ruettger *et al* [14] observed that CatK expression in articular chondrocytes was enhanced by the N-terminal telopeptide of collagen type II via p38MAPK pathway, but not through JNK pathway. Collectively, these studies strongly suggest that CatK expression is tightly regulated by MAPK pathway. Nevertheless, the incomplete blocking of UVA-induced CatK expression by MAPK inhibitors might suggest other signaling pathways involved.

Since activation of MAPK pathway enhances transactivation of AP-1, we hypothesize that AP-1 may play a role in the regulation of UVA-induced CatK expression. Analysis of real-time RT-PCR and western blot revealed that knockdown of Jun and Fos significantly decreased both basal and UVA-stimulated CatK expression at transcriptional and protein levels (Fig. 6C and 6D), indicating that AP-1 participates in the regulation of basal and UVA -induced CatK expression. Similar to our findings, members of the AP-1family have been reported to regulate CatK transcription in RAW cells [32]. A mouse homologue of Jun dimerization protein 2 activates CatK promoter in transient transfections of mouse preosteoclast RAW cells [33]. Moreover, Pang *et al* [18] noted that siRNA targeted against c-Jun or JunB significantly inhibited CatK mRNA expression in response to RANKL, and proposed that the promoter region of CatK gene might contain potential AP-1 binding sites. However, Sharma *et al* [34] described an AP-1 independent interaction of transcription factors at ∼1500 bp of CatK promoter, suggesting that regulation of different promoter regions may interact to regulate CatK gene expression in osteoclasts. Further studies are needed to clarify the mechanism underlying the response of CatK promoter to AP-1 and whether AP-1 directly binds to CatK promoter and/or interacts with other nuclear factors to enhance CatK transcription in fibroblasts.

The limitation of the present study is that we didn't investigate the effect of cell density on CatK expression. CatK expression is found higher in the confluent skin fibroblasts than the exponentially growing fibroblasts [7], and is much more increased on days 3–7 than day 2 after cells reaching confluence [35]. Since cell density would be increasingly higher at later time points, we seeded the same number of cells to the same size culture plates, and used cells seeded to plates at the same time for the each time experiment to exclude the effect of cell density on CatK expression. Considering that UVA irradiation induces both cell death and cell cycle arrest [36], it is likely that UVA might decrease CatK expression. However, in accordance with previous study [11], the present study showed that UVA increased CatK mRNA and protein expression. One possible explanation is that UVA-induced CatK expression may be regulated more by MAPK/AP-1 pathway than cell density. Nevertheless, the exact mechanism remains to be studied.

In conclusion, our study revealed that UVA could increase CatK expression early after irradiation in fibroblasts, which could be attenuated not only by inhibition of JNK and p38 MAPK pathways, but also by knokdown of Jun and Fos. This suggests that MAPK pathway is probably involved in UVA-induced CatK expression through upregulation of AP-1 activity. The breakdown of dermal collagen in photoaging has been documented to be associated with UV-induced MMPs expression, which is also regulated by MAPK/AP-1 pathway [37] [38]. Currently, some anti-photoaging agents are chosen according to their ability to decrease MMPs expression through inhibiting UV- activated MAPK pathway. Based on our data, such agents, however, might increase the risk of decreasing CatK expression, which will contribute to solar elastosis. Our finding presents a new possible molecular basis for anti-photoaging therapy.
